# Supplementary material for: A Bibliometric Analysis of Research on the Role of BDNF in Depression and Treatment
Source: Biomolecules. 2022 Oct 12;12(10):1464. doi: 10.3390/biom12101464 (PMC9599058; doi:10.3390/biom12101464)
Supplement: Supplementary file 1 [file biomolecules-12-01464-s001.zip › biomolecules-1929255-supplementary.pdf]

Supplementary

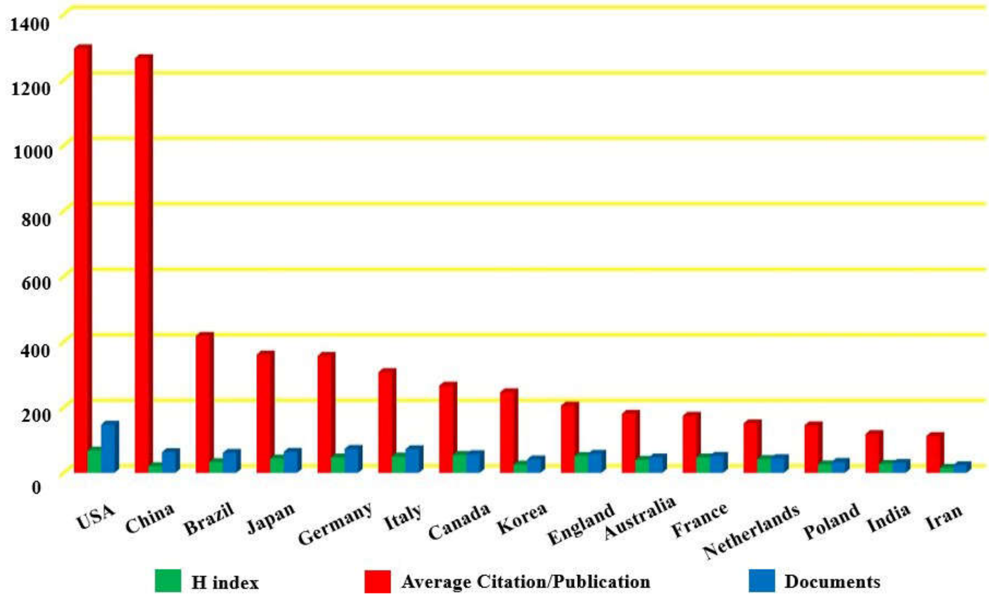

Figure S1. Contributions of countries or regions

The total number of publications counts, average citation per item, and H index of the top 15 countries.

Keywords	Year	Strength	Begin	End
messenger rna	1999	35.42	1999	2008
nerve growth factor	1999	21.61	1999	2009
long term potentiation	1999	13.36	1999	2007
dentate gyrus	1999	16.07	2000	2012
factor bdnf	1999	11.97	2000	2009
rat brain	1999	48.53	2001	2009
element binding protein	1999	20.98	2001	2011
rat hippocampus	1999	17.6	2001	2011
antidepressant treatment	1999	13.81	2001	2009
mood disorder	1999	10.91	2002	2010
trkb messenger rna	1999	10.36	2002	2010
family based association	1999	21.88	2004	2010
bipolar disorder	1999	15.22	2004	2009
factor messenger rna	1999	12.61	2004	2009
cell proliferation	1999	11.13	2004	2013
depressed patient	1999	16.26	2005	2012
human memory	1999	15.21	2005	2015
activity dependent secretion	1999	18.01	2006	2015
human platelet	1999	12.14	2006	2012
neurotrophic factor bdnf	1999	11.25	2006	2010
factor val66met polymorphism	1999	10.41	2008	2014
oxidative stress	1999	15.55	2018	2022
mechanism	1999	12.38	2018	2020
neuroinflammation	1999	12.34	2018	2022
inflammation	1999	11.27	2018	2022

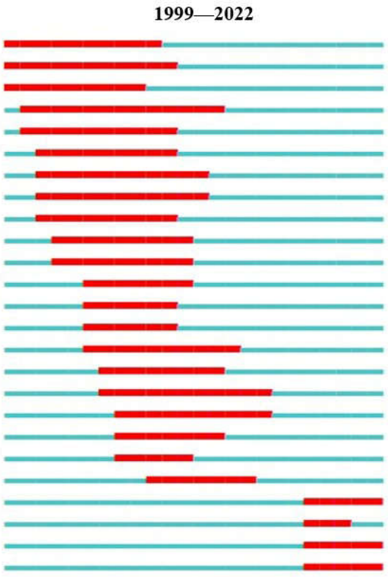

Figure S2. Keyword analysis

Top 25 keywords with the strongest citation bursts on the association between depression and BDNF according to CiteSpace.

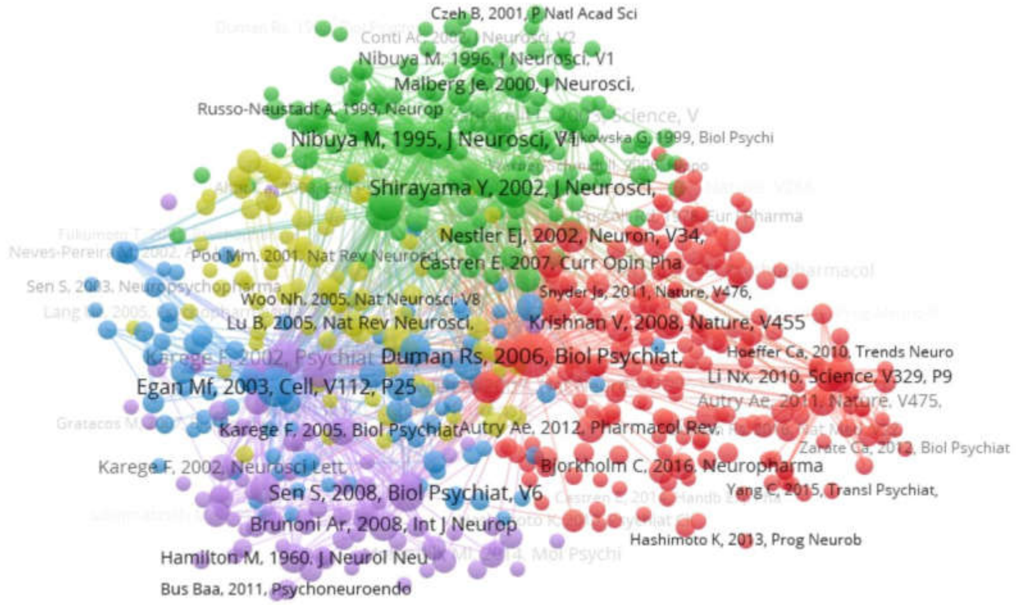

Figure S3. Cited references analysis

Mapping of the co-citation analysis among the citied references which occurred at least 39 times on the association between depression and BDNF according to VOSviewer. Node lines represent association strength. Node colors represent different clusters.
